# Exploring the experience of family caregivers of children with medical complexity during COVID-19: a qualitative study

**DOI:** 10.1186/s12887-023-03944-z

**Published:** 2023-04-06

**Authors:** Natalie Pitch, Laura Davidson, Samantha Mekhuri, Richa Patel, Selvi Patel, Munazzah Ambreen, Reshma Amin

**Affiliations:** 1 Division of Respiratory Medicine, Department of Paediatrics, The Hospital for Sick Children, 4539 Hill Wing, 555 University Ave, Toronto, ON M5G 1X8 Canada; 2 Department of Paediatrics, University of Toronto, Toronto, ON Canada; 3 Child Health Evaluative Sciences (CHES) SickKids Research Institute, Toronto, ON Canada

**Keywords:** Children with medical complexity, Family caregivers, Caregiver burden, COVID-19 pandemic

## Abstract

**Background and objectives:**

Children with medical complexity have been disproportionately impacted by the COVID-19 pandemic and the associated changes in healthcare delivery. The primary objective of this study was to gain a thorough understanding of the lived experiences of family caregivers of children with medical complexity during the pandemic.

**Methods:**

We conducted semi-structured interviews with family caregivers of children with medical complexity from a tertiary pediatric hospital. Interview questions focused on the aspects of caregiving for children with medical complexity, impact on caregiver mental and physical well-being, changes to daily life secondary to the pandemic, and experiences receiving care in the healthcare system. Interviews were conducted until thematic saturation was achieved. Interviews were audio recorded, deidentified, transcribed verbatim, coded and analyzed using content analysis.

**Results:**

Twelve semi-structured interviews were conducted. The interviews revealed three major themes and several associated subthemes: (1) experiences with the healthcare system amid the pandemic (lack of access to healthcare services and increased hospital restrictions, negative clinical interactions and communication breakdowns, virtual care use); (2) common challenges during the pandemic (financial strain, balancing multiple roles, inadequate homecare nursing); and (3) the pandemic’s impact on family caregiver well-being (mental toll, physical toll).

**Conclusions:**

Family caregivers of children with medical complexity experienced mental and physical burden due to the intense nature of their caregiving responsibilities that were exacerbated during the pandemic. Our results highlight key priorities for the development of effective interventions to support family caregivers and their children.

**Supplementary Information:**

The online version contains supplementary material available at 10.1186/s12887-023-03944-z.

## Introduction

Raising children amid a pandemic is challenging for all caregivers but may be particularly difficult for those caring for children with medical complexity (CMC). These children have multiple chronic conditions, significant functional limitations, and dependence on technology [1, 2]. While CMC comprise 1% of all children, the size of this population is rising due to advances in medical care [3, 4]. CMC use the healthcare system frequently and intensely. However, a significant proportion of care for them is provided in the home by family caregivers (FCs) [3, 5].

A study by Verma et al. [6] revealed that the prevalence of psychosocial stress in FCs of CMC is amongst the highest of all studied pediatric populations. Due to the complexity of their care needs, CMC were likely disproportionately impacted by the COVID-19 pandemic and associated changes in healthcare. CMC experienced decreased access to allied health services and medical specialists, with the long-term impacts unknown [7]. There were also disruptions to the delivery of homecare nursing, a long-standing challenge already faced by FCs [8, 9]. Further, there was a significant shift to virtual care to mitigate virus exposure [10]. There is a critical need to understand how pandemic-related changes have impacted the caregiver burden of CMC from the perspective of FCs [11, 12].

To our knowledge, the impact of the pandemic on FCs of CMC has not yet been explored qualitatively and this could provide important insight into their lived experiences. Our primary study objective was to gain an improved understanding of the caregiving experience for CMC during the pandemic. We also aimed to provide relevant stakeholders with recommendations from FCs on how to best support their ability to care for their children and family.

## Methods

### Study design

We conducted qualitative interviews with FCs of CMC between November 2021 and January 2022. Institutional research ethics approval (ethics #1000057112) was obtained from the Hospital for Sick Children (SickKids). Participants were purposively selected from a cohort of 91 FCs who completed the Psychosocial Assessment Tool (PAT) as a part of another study (Ethics #1000057112). The PAT is a validated, short self-reported screening tool used to assess psychosocial risk in caregivers of pediatric patients [13]. The total PAT score assigns FCs to one of three levels of psychosocial risk: low-risk ‘Universal’ families with normal transient levels of stress (total score < 1.0), intermediate-risk ‘Targeted’ families with acute or elevated levels of stress (total score between 1.0 and 1.9) and high-risk ‘Clinical’ families with severe stress (total score ≥ 2.0) [14]. The inclusion criteria were: (1) FC of a child less than 18 years with medical complexity defined as having complex chronic conditions and/or neurologic impairment requiring specialized care, substantial healthcare needs, functional limitations and high healthcare resource utilization [1]; (2) followed in the SickKids Long-Term Ventilation clinic (3) scored in the ‘Targeted’ or ‘Clinical’ risk category. We intentionally recruited participants from these two categories to explore the impact of the pandemic on the experiences of FCs who are at highest risk of psychosocial stress. Purposive sampling of FCs was used to ensure representation of CMC at various ages, diagnoses, healthcare needs, caregiver experiences and PAT scores. Eligible families were identified and introduced to the study by a clinical social worker who previously completed their social work assessments. Written informed consent was obtained by a research coordinator prior to initiation of the interview.

### Data collection

Data collection included online demographic surveys and single semi-structured interviews. The interview guide was developed by the research team following a review of the relevant literature and consultation with content experts. All interviews were conducted in English. After the first three interviews, transcripts were reviewed by members of the research team. Revisions were made to the interview guide to improve clarity and additional questions were added based on the content (see Additional file 1, for the interview guide). Interviews were 40 to 90 min long and conducted virtually using a video conferencing platform (Zoom Video Communications, San Jose, California) by a female social worker with formal training in qualitative research (LD). Participants were given a nominal gift card as a token of appreciation. Transcripts were not returned to participants; however, they were provided a description of the coding framework. The Consolidated Criteria for Reporting Qualitative Research checklist was used to guide the study methods and reporting (see Additional file 2) [15].

### Data analysis

Interviews were audio-recorded, deidentified and transcribed verbatim. Data analysis software (NVivo version 12 [QSR]) was used for data management and coding. Four-step content analysis was conducted to identify, code, and categorize predominant themes from the text. After an immersive reading of the transcripts by 4 study investigators (NP, MA, LD and RA), initial patterns and recurring categories were identified [16]. Next, similarities and differences between participant accounts were identified. Finally, codes were created by 3 study investigators (NP, MA, LD). A codebook was created and iteratively modified by 4 study investigators (NP, MA, LD and RA). Methodologic rigor was established through prolonged engagement and peer debriefing. After 12 interviews, recruitment was considered closed because the authors agreed that data saturation was achieved, defined as the point when additional data did not lead to the emergence of new themes [17].

## Results

### Study sample

Sixty FCs met the inclusion criteria for participation. The social worker contacted 23 FCs via phone or e-mail for consent following the completion of their social work assessments. Overall, 4 (17%) were unable to be reached, 4 (17%) declined participation; thus 15 (65%) FCs agreed to participate. Of the 15 consented participants, 12 completed the interviews. The remaining three had ultimately not committed to an interview time before data saturation was reached. See Table 1 for FC and child demographics.

**Table 1:** Demographic information of family caregivers and their children

	N (%)
**Caregiver gender**	
Female	11 (91.7)
Male	1 (8.3)
**Child gender**	
Female	4 (33.3)
Male	8 (67.7)
**Caregiver age (years)**	
30–39	5 (41.7)
40–49	5 (41.7)
50–59	2 (16.6)
**Child age (years)**	
< 6	1 (8.3)
6–12	7 (58.4)
13–18	4 (33.3)
**Child primary diagnosis**	
Musculoskeletal disease	8 (66.7)
Central nervous system disease	1 (8.3)
Respiratory disease	2 (16.7)
Unclassified	1 (8.3)
**Annual gross household income**	
$100,000 or more	7 (58.3)
$50,000–$79,999	2 (16.7)
$20,000–$49,999	2 (16.7)
Did not disclose	1 (8.3)
**Employment status**	
Full-time	3 (25.0)
Part-time	4 (33.3)
Unemployed	2 (16.7)
Receiving support	3 (25.0)
**Financial difficulty**	
No problems	4 (33.3)
Some problems	7 (58.4)
Many problems	1 (8.3)

The overarching themes in our study were organized as follows: (1) experiences with the healthcare system amid the pandemic; (2) common challenges during the pandemic; and (3) the pandemic’s impact on FC well-being.

#### Experiences with the healthcare system amid the pandemic

##### Lack of access to healthcare services and increased hospital restrictions

FCs depend on several community and health services such as speech language pathology, occupational therapy, and physiotherapy to meet the needs of their children. However, FCs noted a lack of access to these services as a direct result of the pandemic. These services were either not available or FCs did not want to put their children at increased exposure to the virus. Several FCs consider these services critical to their children and worry that lack of participation in these activities will hinder their development.

Many FCs also described how the implementation of visitation policies only allowing one parent in the hospital at a time during the pandemic made attending hospital appointments extremely difficult. While FCs recognized that precautions are necessary, they believe these policies did not consider the needs of CMC. FCs detailed how attending hospital appointments is more than a one-person job as it involves traveling with the appropriate equipment and facilitating transfers, all while attending to their child’s regular care needs. The need to fight for more supports during hospital appointments made an already challenging situation even more stressful during the pandemic.

##### Negative clinical interactions and communication breakdowns

Many participants described having negative interactions with healthcare providers both in the hospital and in primary care settings. During the pandemic, FCs recalled experiences with healthcare providers who demonstrated insensitivity toward their children. FCs felt these healthcare providers did not make appropriate efforts to develop a connection with their child or adapt their approach in healthcare appointments to accommodate their child’s specific needs (e.g., attending to non-verbal cues in children who are non-verbal). Some FCs felt physicians had a condescending attitude and did not acknowledge their expertise in their child’s care. One FC related an experience in which a nurse did not acknowledge her child and inserted a rectal thermometer without warning, causing the child immense discomfort.

Further, several FCs discussed the challenges of communication with healthcare providers during the pandemic. One FC noted having significant difficulty connecting with her pediatrician, making multiple attempts to get in touch with no response for a long period of time. Another FC recounted feeling let down by her healthcare team when her son’s surgery was delayed due to the pandemic, and she did not receive adequate communication about the ongoing management plan.

##### Virtual care use

Several FCs reported the benefits of virtual healthcare use implemented during the pandemic. They appreciated being able to attend medical appointments through virtual teleconferencing platforms, eliminating the burden of physically going into hospital. This was especially highlighted by FCs who lived far from the hospital. Some FCs with children dependent on respiratory technology highlighted the value of a health app that provided them direct access to healthcare providers. They were able to share data from their child’s technology with their healthcare team and ask questions that could be immediately addressed through the app. This provided FCs with a sense of connectedness to their healthcare team, despite feeling isolated during the pandemic.

#### Pandemic’s impact on FC well-being

##### Physical toll

FCs received less or no nursing support due to the pandemic, adding significantly to their workload. Fulfilling the chronic and intense care demands of CMC was described as physically exhausting. Caregivers provide highly specialized care including medication administration, tube feeding, tracheostomy support, technology management and emergency response. The examples shown in Table 2 of a typical day for a FC highlights the rigorous nature of this care. The demands continue throughout the night, resulting in many FCs experiencing sleep deprivation, especially when nursing support is not present. Moreover, caregivers described the physical challenges associated with transferring their children who are physically impaired from their disease. The cumulative effect of repeated lifting and transfers takes a physical toll on FCs, especially as their children develop. One FC highlighted how the pandemic worsened the physical demands of attending hospital appointments, as the one-visitor policy prevented her from having support with transfers during her visit.

**Table 2:** Themes and subthemes with illustrative quotes

**Experiences in the healthcare system amid the pandemic**	Lack of healthcare services access and increased hospital restrictions	“It’s quite hard in the hospital setting as well, you know, getting appointments, taking her alone. When she has an appointment, it’s really hard because they only allow one caregiver to go with her. This is the worst thing for me because I plan to manage 2-3 appointments in 1 day. There is an upcoming appointment next Tuesday, so 12 o’clock there is long-term ventilation clinic appointment, the second is at 2 o’clock and it is neurological, then VNS appointment. In between, they asked me to do the x-rays for her and bone density because she’s due for endocrinology because she has disuse osteoporosis, and they want to monitor her. So you can imagine and she’s big. I have to transfer her every time. I have to change her. I have to do everything. I’m thinking about next week like how am I going to manage it because I have to be doing everything all together in one appointment.” “There needs to be a nurse, my husband, and myself, because there’s so much medical equipment, like we’re a mini crash team. So, for safety reasons, we have to be three. People aren’t understanding the magnitude of care that this child needs to keep her safe. I think more needs to be done to understand and support families. It’s a huge form of stress. Every time I come in, I have to advocate every single time for my kid. It’s exhausting.”
	Benefits of virtual care use	“That gave me a sense of connection to the respiratory team. I knew we weren’t actually seeing them which was hard because we’re so secluded in our home. But, having that connection and easy access to them, and them to us, made a massive difference. I didn’t feel like I was all of a sudden by myself.”
	Negative clinical interactions and communication breakdowns	“Also, they don’t treat her properly either. For example, one of our hospital experiences during the pandemic was that a nurse didn’t even acknowledge her, didn’t even talk to her, didn’t even warn her that she was going to stick a thermometer, like a rectal temperature. So she shoved it in there. X had tears coming down her eyes and the nurse was oblivious, had no idea she put my daughter into discomfort. Why wouldn’t you warn her? Why wouldn’t you say, X, I’m going to take your temperature, it might hurt a little bit. Just warn her and prepare her. You would with a typical child, you would definitely do that with a typical child. Why would you not do that for her? Does she not have value enough for you to do that?” “If it was a regular child who could speak then you would treat her differently, but because she doesn’t speak, you’re assuming that she doesn’t understand. So you treat her differently. It’s not fair. It’s not okay. How would you feel if somebody is ignoring you and talking above you?”
**Common challenges during the pandemic**	Financial strain	**Interviewer question:** “Would you say there’s any changes in the last year with regards to your finances during the pandemic, has it increased?” “For sure, because I’m off work. I’m only getting a certain percentage of my salary. I mean, at least my benefits are still there. So it’s covering some of the medical things, but even her medicine, some of it is covered some is not. So we’re paying out of pocket for so many different things. That’s the piece where nobody’s really looking at the big picture of like, what are the parents actually having to fork out? But we have, hundreds of dollars of medical expenses for prescriptions every month, it adds up. So it’d be great if it was covered.” “So hydro is through the roof, we spend a lot of money on hydro, because all the medical equipment is running all the time. But we don’t get a rebate for it, we don’t fit the criteria because we’re not in the right bracket, financially… Again, like you’re maxing out your credit cards, you’re maxing out your lines of credit. So financially, you’re not in a good place because your expenses far exceed everything else because of all the medical needs. She’s so expensive because we’re paying for nursing, and yes, insurance will reimburse us, but we always have to have disposable income to pay large lump sums constantly where it’s tied up for months at a time. Eventually you get reimbursed but again, you always have money tied up. We’re lucky. We have lines of credit and credit cards, but other families don’t have that. They’re in even dire straits.” “But the burden of finances isn’t just a burden of finance, the fights that my husband and I had was about, did we fill out the paperwork? Did you get the insurance money back? Did you make sure that you kept that receipt from that mundane supply that you bought at X, because the only way we’re going to get money back is if we have the receipt and filled out the paperwork. So, things other families would just, you know, like diapers, for example. There’s a diaper support, and there’s funding to support it, but you have to track every single box of diapers and wipes that you ever bought. The second that you don’t, they send you a document that says, we’re auditing you where are all your receipts for your diapers… You should see us filling out our paperwork to get money back, we have an entire kitchen table filled with papers and receipts and documents that we have to send to four different agencies, you know. We have to send it to the federal government, we have to send one to their insurance. It’s ridiculous.”
	Balancing multiple roles	“Usually, most days, I’m a respiratory therapist. I do homeschooling or virtual school. I’m her nurse, her caregiver, her mother, and her administrator. I’m her advocate… Then, I’m a mother, a wife. That’s over 10 hats that I have on every day. Plus, I used to work outside of the home. I shouldn’t have 10 hats, though. To ask me because I’m her mother to be supermom is not sustainable. I’m her mother, I shouldn’t be her nurse and all those other roles.” “Keeping him safe, keeping ourselves safe, and not having people enter the home is really hard. I’m a very family-oriented person. I’m very close to my mother and father, but because of everything that is going on with the pandemic, I couldn’t have them come to the home. I couldn’t have my sister, brother, and uncles. I’m sure my older son probably had a hard time because he couldn’t have his friends come over either. I think that was the most difficult thing, keeping him safe during this pandemic, and not having nursing and me and my husband alternating a 24-h shift together.” “My days are completely full. I don’t know from 1 day to the next whether a nurse will be present or not. Even if a nurse is present, we’ve lost a lot of experienced nurses. Now, we’ve onboarded a lot of newer people, we have a call bell that gets pressed daily. For emergencies, we’re like a mini crash team where we have to basically mobilize, come down, and help and support. During the day, I have zoom calls with the hospital, there’s multiple teams involved, lots of coordination of care, prescriptions to be ordered, medical supplies, equipment, and trying to order in repairs. There’s so many moving pieces. For paperwork, I have claims to fill out, apply for funding, schedule nurses, bill nurses, train nurses, like every day is a full day. So, by the time the day ends, I’m exhausted or I’m crashing. Every day is full like you can’t turn it off.” “On top of all that, I’m doing advocacy. A group of us got together came up with nine recommendations to help with the homecare crisis, have been trying to get through the front door to the Ministry of Health since September. We’re not getting anywhere, that’s where it’s so frustrating. As parents, we have possible solutions to help with the major problems happening. But we can’t even be heard because we’re not in a position or authority… I’m a real person, I’m struggling, I’m calling out. It’s a lot for me to even ask for help or to come forward, because my life is so chaotic, to not be able to get through the front door is really frustrating for me.” “… you’re always fighting and advocating for your child. It’s silly things like, the schools don’t always have working elevators. I work in a separate school, and we don’t have a working elevator, like basic human needs are not readily available for children like X. So those are things, like I shouldn’t have to go to weekly meetings, I shouldn’t have to fight to get the basic needs for my child. Those are the other things on top of being a mom, the working, the being a nurse, like other things you shouldn’t have to go through which exhausts a person.” “Well, there’s no end in sight. For me, I know that there’s nothing in the pipeline, the nurses that I have are the nurses that I have. I called so many agencies to try to hire other nurses. I’ve got so many barriers around me. But I’m not giving up. It just takes so much out of me everyday to advocate for supports… It’s the same pool of people that all of us are competing for the same people. That’s where it gets frustrating because there’s nothing I can do to improve my situation or my lot. I’m not going to give up, which is why I’m advocating because if nothing changes, we’re going to be in worse shape down the road. I’ll be in worse shape down the road. So, while I have a little bit of energy, I feel like important to speak up right now.” “So, all my attention is only on X. I do have a 19-year-old son. He has been through a lot, obviously, with X and then having a brother with this condition. He loves him to death, but we tend to just focus on him and not really focus on the 19-year-old even though sometimes he needs us. That’s a stress alone by itself because he’s dealing with his own things, and we don’t know how to meet his needs because we’re dealing with our own things.” “Also, it was really hard on our daughter. She’s older, and we would go to the hospital and leave her at home and didn’t realize that she was not feeling a part of things and kind of left out. I learned that along the way, but I mean, she was always left behind. So, it was tough to be in two places at once. “
	Inadequate homecare nursing	“I was in deep sleep… I’m hearing her alarms going off. … she was bawling her eyes out screaming for the nurse. I go downstairs, and she’s inconsolable. She’d been screaming for the nurse for about 45 min. I go to where we have a nursing station set up in our basement where my daughter’s room is. … the nurse is asleep on our couch which is around the corner. I literally just woke her up and said time for you to go home now because if that was an actual true emergency…” “Well, and with COVID and now with what’s happening with the nursing system collapsing, all the resources were taken out of homecare to go into hospital and long-term care. But there hasn’t been any influx of new nurses coming into homecare. So, what is there is bare bones. It’s hard to retain staff, it’s hard to onboard staff. While we were in the hospital last time, we lost five nurses. So, it’s like having to onboard new people that don’t have the right skill set, and just plugging in holes. But, that takes a toll because you’re always on because they don’t have the skill sets to take care of her. So you need an ICU nurse, but you’re getting mental health nurses and nurses that are very inexperienced who can’t clear her airway and you do most of the tasks required for her daily care.” “During the pandemic, not only did we have nurses call in sick at the last minute, because they might not have passed the screening or they had a sniffle, but we also had nurses leaving us. It’s much better to do day shift or doing tests at a testing… We had nurses that had to isolate because they were caring for their elderly parents so they left work because they couldn’t be exposed. But it was learning about that at the last minute. So like, if a nurse was on shift, and then they quit, there was no two weeks notice during the pandemic. There’s no two weeks notice on being sick or having symptoms. So, we were finding this out constantly, shift after shift, week after week, after working a full day caring for my child on my own.” “You just barreled through, there was no preparation, there was no result. During the pandemic, there wasn’t even a thought process. Pre-pandemic, if a nurse called in sick on a Friday night, I would say, can we get a nurse in during the day on the Saturday, which we normally wouldn’t have, but it would give us some time to sleep, right? Or, can we get a nurse to stay an extra two h in the morning just so I can sleep the next night. But that didn’t exist in the pandemic because we just wanted a nurse. So, I wouldn’t even rock the boat by asking for anything because I didn’t want to potentially ruin the ability to get a nurse.” “Then, the real frustration is that you’re not really dealing with the person that’s in charge of it. So because of that, you have no way to hold people accountable. Like if I go to the hospital and my doctor misdiagnoses me, I can hold that doctor accountable by complaining and speaking to them about that mistake. With nursing, if somebody doesn’t show up or quits and doesn’t give notice, I can’t call like there’s nothing we can do. Such an important part of managing complex care is having people that you can trust, that are consistent with the way in which you sort of do things. As parents, we must lower our standards just to keep people here, so that we can sleep and be productive human beings. I was at the ministry when talking about some other aspects of my daughter’s care and the cost of treatment and so on. I know that in the calculus, they really don’t put a high enough cost on the lost productivity of the caregivers when care is offloaded onto the caregiver. So when care is offloaded onto me, the amount of lost productivity is incredibly high. Earning potentially drops, there’s a bunch of things that drop, and then there’s a bunch of other knock-on effects that impact.”
**Pandemic’s impact on FC well-being**	Physical toll	“I have to wake up around 7:00-7:30 to give her the medication before starting her feed. I have to start at 8 so it can finish at 12. I have to change her, dress her up, put her on the wheelchair, everything. Before starting the feed, I do the mouth care. We have to give her the Ventolin puffs, and then start her feeds. Then, around 12:00 pm, when she finished her feed I bring her out and I have to change the diaper again. I cook that time… Meanwhile, I have other daughter also. So, leaving her at daycare and everything. Then, before 2:00, I have to give her the medication again. So, 2:00 we start her feeds to roughly around 5:30-6:00. We have to finish her feed so we can give her 8:00 medications, again, in between we have a couple of medications. Then, 8:00, we start her feeds and close it around 11:00. Then half an hour gap we give her for water because she’s not catching up the water. We have to start at 11:30 after changing the diaper. So, around 12 am my routine is finished with her.” “Well, thank god that my husband wakes up early. He would get up early and give him his medication at 8:30. Then, he would do cough assist at 10:00 and he would have to give back meds at 12:00. By 1:00, I’m usually up because I sleep late in the night. So, I wake up at one, and I will do his meds at 3:00. I will do cough assist at 4 and I will give him his meds at 6:00… So then my husband will have him at 7:00, at 8:00 give him his back meds and do another cough assist. Then, he would say, okay, M, it’s your turn. You’re going to sleep with him tonight. He will watch him from 6:00-11:00. Then, at 11:00, I will take over, go in the room with him and just be with him.” “One thing, and I know that it’s for our safety that the hospital did the caregiver thing, but people like X need to have exceptions. To have more than one caregiver to come for appointments, because lifting X and even sometimes it’s hard for me to change her diaper. Now, we have to do the bone scan and x-rays, I’m the one who’s doing most of the things and it’s hard myself.” “So in terms of the burden of care, there were tons of things to learn, things to constantly watch out for, and things to be on top of so that you were prepared in case of an emergency. So, we call it a go bag. That’s his bag with all of his care equipment, his suction machine, spare trache kits, all that sort of stuff. So his bag is constantly checked and rechecked to make sure if an emergency did happen, everything was on hand, you know.” “Then, getting ready for bed is a whole other set of routine. So making sure, he has a feed made, hooking up his feed, making sure his ventilation is hooked, all that sort of stuff. Every story that I tell in a practical day, I’m not including and maybe I should is the lifting and carrying the vent, bringing the vent, you know, can I carry the vent? Have I packed everything in enough bags that I’m not carrying loose items everywhere, but how can I plan to carry all this luggage or all this stuff that as one person, I could manage that all by myself, right? How was I going to carry it? So we would take a stroller so I could push everything, but I had to make sure everything fit on a stroller. So all that planning, lifting, carrying is kind of embedded in the story without being explicit.” “You got to capture us in the hospital with bracelets to see how much sleep we’re actually getting, how much rest we’re getting, for taking on the role like we are in the hospital, but I’ve never felt so alone. And so, so unsupported, as it has been since COVID. With the hospital. Yeah, I’m surrounded by medical staff. But I’m still expected to do everything. And it’s like, but how is that sustainable? Like I’m more exhausted going in hospital than and being at home with less supports at home. And I decide that that’s a sad situation. So I dread going in, because I know that I’m going to be ill for a week afterwards and have physical manifestations because my body shuts down because I’m that exhausted from the hospitalization. Because I can’t sleep, when am I going to sleep if nobody’s there to watch over her?” “When the pandemic first hit, our nursing went to hell. So essentially, we couldn’t have the nurses in the home. We were having like 4 days on and 4 days off. So one of us would stay up all night with her, then work, and then sleep. Then, we would switch back and forth. So we did that for almost the whole first year of COVID… But, you know, I can flex my schedule. So it was a little bit easier because of that, it would have been impossible otherwise. Then, of course, all of that tension and stress just makes everything more difficult, right? Everybody’s on edge all the time, but everybody’s bored. It’s been incredibly difficult.”
	Mental toll	“I can’t even grieve and process the fact that my daughter’s dying. I can’t even wrap my head around it. I can’t even think about it because I don’t have time. It’s that piece that people forget, like when do I have time to really process and think about it? I don’t, I’m always in survival mode. I’m jumping in doing whatever needs to be done. So it takes its toll.” “I’m in a tailspin mentally, right? COVID made things worse, I’ve got more roles to take on. I have no supports, and I was crashing.” “So, the 30,000-ft view is that prior to the pandemic, we were still doing a pretty good job of separating our parental roles from the medical needs of X. We could go from one mode to the other, and those things could be separated so that we could have our normal interactions, and also do the medical stuff. I think one of the things that COVID ended up doing because we were 24-h a day, seven days a week locked down, it sort of collapsed those things together. So, we were working and we were nurses all day, right? We prepared all the food, prepared the medicines, did all the medical checks, kept all the charting, did the overnights, and so on. That was difficult and caused a tremendous amount of stress.” “Well, we had to be isolated. So we didn’t have anybody coming in. That’s the whole thing. Whatever supports we had before everything is gone. So even family that used to come sometimes. My mother-in-law would come for a week and help out, cook and clean and help out with the house stuff. She wouldn’t help out with my daughter, but she would help with the daily stuff. That’s one less thing I had to do because I’m still a housewife. I got to do laundry, dishes, all the regular stuff everybody does on top of everything else, right? So, it helps to have at least one less thing to do. She’ll make dinner so that’s one less thing I have to worry about. But they didn’t come because of COVID, everything was locked down. Everybody was not allowed to travel. So, we were on our own the whole time. So that’s why we were even more tired because we really didn’t have any of those supports. Then, as nursing supports dwindled, we were taking on more and more of a caregiver role, well, the nursing role. I shouldn’t say caregiver, it’s actually the nursing role.” “I was a nervous wreck in the beginning. I just thought everything, and everyone had COVID. We locked ourselves in our houses for the first three months. I made my husband do ridiculous things, like as soon as he got in the house, take all your clothes off and throw them in the laundry. It was just ridiculous, terrified in the beginning… So, there was a lot of things combined, but COVID was a baseline. It just tore me apart in the beginning. It’s taken a lot of time and patience for all of us to adjust to this new normal, and eventually, we’ll get there. It took a huge toll on us. Isolation was really tough. It was really hard trusting that people were being considerate enough to let us know, if they were in contact with somebody else. We’ve had a few like, oh my gosh, you know, after the fact and not people being malicious, just not thinking and realizing that that’s what we really need. We need to be that much more careful.” “We would be prepared and when the medication got to a certain level, we would tell the nurses to inform us so that we could go reorder medication, and that was always our way of making sure that we weren’t running out of medication. During the pandemic, even that lead time wasn’t enough because some medications were out of stock, or some nurses were working from home some weren’t. How do I get a prescription renewal when I’m doing a virtual meeting or appointment with my doctor. All those things were not simple but they were organized and we knew how to do them all, but that went up in the air in the pandemic.”

##### Mental toll

Participants emphasized the mental toll that results from supporting their children on a 24/7 basis. Ensuring these intense care demands are constantly met in a timely manner was described as stressful and mentally taxing. FCs undergo chronic stress as CMC can experience life-threatening situations requiring high-stake decisions. One FC described being in constant fight or flight mode, with no opportunity to nurture her own mental health. Many FCs expressed guilt for not doing enough, making poor decisions, or needing extra support. One highlighted the mental toll of witnessing her child deteriorate without having time to process her emotions or grieve.

This underlying mental exhaustion was exacerbated by the pandemic. FCs described being in constant fear of their child or family contracting COVID-19, especially knowing that their child is particularly vulnerable to infections. Some FCs hesitated to bring their child to the hospital in emergency situations. Several FCs had difficulty accessing medications or supplies, causing significant stress. Many FCs implemented strict social distancing measures by limiting outings and visitors (friends and family) in the home. This lack of visitor support left them feeling isolated and exhausted from taking on more demands. Some noted that every day felt the same without the ability to engage in fun activities or self-care. Many FCs described feeling trapped inside their homes, with no separation between their work and home environment. Interestingly, a subset of FCs expressed that the circumstances brought on by the pandemic were similar to what they already experienced due to their child’s chronic illness.

#### Common challenges during the pandemic

##### Financial strain

The financial burden of caring for CMC was worsened for FCs who lost work because of the pandemic, resulting in less income. FCs emphasized the significant costs of caring for CMC due to the need for specialized medical supplies and equipment, therapies, prescriptions, and nursing. FCs acknowledged some benefits of universal health coverage; however, they are still obligated to pay an extraordinary amount of out-of-pocket fees and many components of care do not have coverage. Even if certain purchases are reimbursable, FCs must pay large sums of money upfront and wait months for reimbursement. Some FCs have had to leave full-time jobs to manage care demands, rendering their families one-income households. The burden of administrative work associated with finances included completing paperwork, tracking purchases, filing receipts, applying for funding, and seeking claim reimbursement. Some FCs noted that being in a higher income bracket put them at a disadvantage. The government does not provide these families with as much support; however, the government does not account for their expenses, leaving them without adequate financial assistance.

##### Balancing multiple roles

At baseline, FCs described the need to balance several roles. FCs perform specialized medical tasks including administering medications, managing medical technology, facilitating feeds, and responding to emergencies. They also coordinate their child’s care, which involves organizing appointments, communicating between specialists, hiring and scheduling nurses, and ordering equipment. Prior to the pandemic, FCs leveraged the support of extended family and homecare nursing to help manage these intense care demands. However, during the pandemic FCs noted having decreased access to homecare nursing and limited help from family to mitigate exposure. Thus, the difficulty in balancing multiple roles was intensified following the pandemic, needing to fulfill all their responsibilities with overall less support.

FCs also highlighted the need to act as advocates for their children on an individual and systemic level. The need to be advocates was also intensified by the pandemic. One FC felt distressed from having to fight for her son’s surgery that kept being cancelled due to the pandemic. Despite the ongoing advocacy efforts of FCs, some feel frustrated that their voices are not heard, with little progress being made. With the amount of time dedicated to caring for their sick children, FCs noted difficulty in fulfilling their obligations as a parent to their other children. Some expressed worry that their other children often feel excluded or that their own needs are not being met. FCs who were extremely cautious during the pandemic to protect their medically fragile child felt guilty for preventing their other children from seeing friends.

##### Inadequate homecare nursing

FCs experience challenges obtaining stable homecare nursing, and this was exacerbated by the pandemic. There was significant nurse turnover, resulting in a lack of care continuity that created distress for their children. Additionally, FCs spent a significant amount of time scheduling nurses and finding fill-ins for frequent cancellations. The necessity to use new nurses was even more distressing during the pandemic as FCs wanted to mitigate potential COVID-19 exposures. As a temporary solution to the nursing shortage, FCs accepted support from nurses who lacked the appropriate training. Consequently, some FCs reported not being able to truly disengage from their caregiving role even with nurses present. One FC emphasized the lack of an official body to hold nurses accountable, making him feel powerless to improve the homecare nursing situation.

## Top FC recommendations

FCs were asked what their top recommendations would be to best support families in caring for CMC during the pandemic and in general. The recommendations were as follows:

**Table:** 

1.	Increased access to reliable homecare nurses with appropriate training specific to CMC and managing medical technology
2.	More robust psychosocial support offered to caregivers and children, especially in preparation for and following discharge from hospital
3.	Increased financial coverage or funding for medication and medical equipment
4.	Improved experience of in-hospital appointments and admissions by better accounting for the needs CMC and their family
5.	Promotion of virtual healthcare to improve barriers to care related to distance and time
6.	Opportunities for caregivers of CMC to connect with each other and share experiences
7.	Improved training and educational resources for caregivers whose children require medical technology
8.	Increased support during the transition between hospital and home
9.	Always allow more than one caregiver to accompany CMC during hospital visits or admissions

## Discussion

This is the first qualitative study to our knowledge to investigate the impact of the COVID-19 pandemic and associated changes in healthcare on FCs of CMC. The results highlight key challenges faced by this population that were already well recognized, with the pandemic now exacerbating them further. Additionally, as the world recovers from the pandemic, these families are now being left in an even more vulnerable position. We provide relevant stakeholders with recommendations not about preparing for a future pandemic response but rather for systemic changes needed now. The pandemic experience has elucidated the health system limitations for CMC and their family caregivers.

Our study highlighted the lack of access to reliable homecare nursing during the pandemic. Homecare nurses are critical to the care of CMC, allowing necessary respite for FCs to take breaks, sleep and maintain some semblance of work-life balance [18, 19]. Even before the pandemic, families faced many barriers in accessing trained homecare nurses [9, 20–22]. Previous studies have documented an association between less support from night nurses and adverse outcomes, including sleep deprivation and symptoms of depression [23, 24]. Our study highlights the profound impact a lack of stable homecare nursing has on FC overall well-being. It is thus not surprising that the most common recommendation from our participants was to improve the overall reliability and accessibility of homecare nursing. One solution might be to encourage the use of unregulated respite care, which has been shown to offer unique advantages in this population [25]. Strategies to achieve this on a more systematic level could include better integrating homecare into nursing education and decreasing the pay disparity between homecare and medical facilities. While this may seem like an expensive approach, these long-term solutions may mitigate the need for acute care services ultimately more costly to our system [26].

The financial burden of caring for CMC is significant, with almost 70% of parents of CMC reporting financial hardship [27]. Although our study was conducted in the context of a publicly funded healthcare system, our study highlights a critical gap for FCs of CMC. Due to the enormous costs associated with caring for CMC, even families earning middle-class incomes face significant financial hardship. Healthcare providers should advocate on behalf of their patients for candidacy for medical coverage to be considered in the context of the significant costs for medications and nutrition, technology supplies, nursing services and equipment required to meet the needs of CMC. Further, processes to obtain funding and reimbursements should be streamlined to mitigate the burden placed on FCs.

CMC spend a significant amount of time in the healthcare environment given their need for intense monitoring and frequent health crises requiring emergency care or hospital admissions [3]. In line with our findings, previous studies have reported the frustration felt by parents when medical providers do not recognize their expertise in their child’s care [28, 29]. Our study adds to this literature in that FCs reported dissatisfaction with how physicians acted towards their children. Evidently, there is a critical need for physicians to adapt their approach to patient care to meet the specific needs of CMC to strengthen their relationship with the child and, in turn, with their family. Parents of CMC appreciate when healthcare staff prioritize getting to know their child and family well [28]. The partnership between healthcare staff and FC is a key component of family-centered care, an approach that optimizes the delivery of care for CMC [29–31].

A component of care for CMC emphasized as a stressor by our sample was a lack of support during hospital experiences, highlighting the need to optimize conditions during hospital visits and admissions. A study by Diskin et al. [32] described how changes in hospital measures implemented during COVID-19 impacted the delivery of healthcare in a pediatric inpatient unit. Visitor restrictions resulted in family separation and lack of support, which was challenging for families during high-stress periods. In another qualitative study examining  the implications of pandemic safety measures, parents of seriously ill children noted feeling more isolated and burnt-out during hospital admissions and clinic visits due to visitor restrictions in medical facilities [33]. In keeping with these findings, several FCs in our study highlighted the difficulties in attending in-hospital appointments as a sole caregiver and recommended allowing more than one caregiver to accompany CMC in hospital at all times.

Despite the barriers created by COVID-19, the use of virtual technology to meet care requirements while maintaining social distancing restrictions was beneficial for our cohort. This finding aligns with other studies demonstrating the value of virtual care and telemedicine in the care of CMC and other paediatric populations [10, 33, 34]. The use of virtual healthcare can mitigate geographical barriers and financial costs associated with in-person visits such as parking fees and gas expenses. Further, eliminating the need for CMC to be in the hospital environment mitigates their risk of acquiring illnesses.

Our study highlighted the immense physical and mental exhaustion endured by FCs raising CMC during the pandemic. Worsening mental health outcomes in FCs of other pediatric populations during the pandemic have also been reported [35–39]. Consistent with research conducted before the pandemic, FCs in our study reported sleep deprivation, mental health challenges, physical exhaustion, and burnout [23, 40, 41]. Clearly, FCs of CMC experienced a unique burden during the pandemic that has not been adequately addressed by the healthcare system. Edelstein et al. [42] outlined promising interventions to reduce stress in FCs of CMC, including care coordination models, respite care, peer support groups, telemedicine, and other health-related supports. The authors suggested that relevant stakeholders should consider combining interventions at the individual and health-systems level to ensure the unique needs of CMC and their FCs are met. In the current landscape of the pandemic where demands placed on caregivers are intensified, further development and implementation of interventions to address issues created by the pandemic is critical to supporting FCs of CMC.

There were some notable limitations to this study. Firstly, our FC sample was recruited from a single tertiary institution, meaning our results may not generalize to FCs of CMC internationally. However, the findings of our cohort do seem to resonate with the published literature for FCs of CMC. Secondly, we only included FCs with intermediate or high levels of psychosocial risk but not low-risk FCs. We intentionally recruited participants from these two categories to explore the impact of the pandemic on the experiences of FCs who are at highest risk of psychosocial stress. Thirdly, our sample was comprised predominantly of English-speaking female participants from middle to high-income households. While our sampling strategy was intended to capture CMC with various demographic and clinical factors, our results may not reflect the diversity of FCs serviced by SickKids’s long-term ventilation program. Finally, we used a question directly from the PAT to characterize the financial difficulties of our study cohort: 1) no problems, 2) some problems, and 3) many problems. However, the use of this tool during the pandemic has not been validated, and thus, it is difficult to interpret the meaning of these responses.

## Conclusions

Our findings indicate that the pandemic intensified challenges already faced by FCs of CMC, increasing overall burden. Further, healthcare measures implemented to mitigate the spread of COVID-19 significantly impacted the way in which CMC received care. FCs provided important recommendations including more accessible homecare nursing, enhanced psychosocial services, and increased financial support. Healthcare policy makers and government stakeholders should consider these meaningful insights when developing interventions targeted toward supporting this medically fragile population and their FCs.

## Supplementary Information

**Additional file 1.** Qualitative Interview Guide.

**Additional file 2.** COREQ (Consolidated criteria for reporting qualitative research).

**Additional file 3.** Initial Coding Tree.

## Data Availability

The datasets used and analyzed during the current study are available from the corresponding author on reasonable request.

## Abbreviations

CMC: Child/children with medical complexity
FC: Family caregiver
