# Supplementary material for: Exploring the experience of family caregivers of children with medical complexity during COVID-19: a qualitative study
Source: BMC Pediatr. 2023 Apr 6;23:160. doi: 10.1186/s12887-023-03944-z (PMC10077324; doi:10.1186/s12887-023-03944-z)
Supplement: Supplementary file 1 — Additional file 1. Qualitative Interview Guide. [file 12887_2023_3944_MOESM1_ESM.pdf]

Additional file 1: Qualitative Interview Guide

1. Can we start off with you telling me about your child and their special care needs?
    a. How have your child's needs affected you as a parent (prompt: mentally, physically?)

2. Can you tell me a story that reflects an average day in your life caring for your child/children?
    a. On a day like this, how would you describe your stress levels and ability to manage the burden of care?
    b. How are you able to incorporate coping mechanisms into your everyday life?
    c. How do you feel about being able to meet all of the needs of your child? What is the biggest barrier?

3. Now can we talk about how the other adults that also care for you child. Can you tell me about these relationships?
    a. Are these supportive?
    b. Any sources of stress?

4. Money is a concern for many people. Is this something you worry about? Can you tell me about this?
    a. Have your child's care needs affected this?

5. The pandemic has changed all of our lives. Can you tell me about how the pandemic has affected your life as a caregiver and what adjustments were made in order to best support yourself and family?
    a. How are you feeling about these changes in regards to your emotions and behaviors?
    b. What has been the biggest challenge of the pandemic for you?

6. Can you tell me about a really bad day during the COVID-19 pandemic? Maybe where you had a medical emergency or experienced shortage in nursing staff?
    a. Can you tell me about this day and how you were feeling?
    b. Can we talk about your experience with these demands and stresses?
    c. What resources if any helped?

7. Can you tell me about your support network as a caregiver during the COVID-19 pandemic?

8. What about resources outside of your family and friends? Can you tell me about those?
    a. Do you have access to the supports you need? Can you tell me about them?

9. Have you ever used any mental health resources (prompt: before, during the pandemic)? Can you tell me about that experience?
    a. What effect did it have on how you were feeling?
    b. If no, have you ever considered speaking to a mental health professional?

10. If you could share one lesson learned with healthcare providers caring for children like yours what would it be?

11. If you had a magic wand to overcome any barrier to care you are experiencing as a caregiver, what would it be?

12. We are coming to the end of the session, is there anything else you'd like us to know, that you haven't had a chance to share?
