# Supplementary material for: Exploring the experience of family caregivers of children with medical complexity during COVID-19: a qualitative study
Source: BMC Pediatr. 2023 Apr 6;23:160. doi: 10.1186/s12887-023-03944-z (PMC10077324; doi:10.1186/s12887-023-03944-z)
Supplement: Supplementary file 3 — Additional file 3. Initial Coding Tree. [file 12887_2023_3944_MOESM3_ESM.pdf]

**Additional file 3: Initial Coding Tree**

**EXPERIENCES WITH THE HEALTHCARE SYSTEM AMID THE PANDEMIC**

- Lack of access to healthcare services and increased hospital restrictions
  - Lack of access to allied health
  - Decreased participation in supportive therapy
  - Challenges of in-hospital visitor restrictions
  - Other
- Negative clinical interactions and communication breakdowns
  - Feeling disrespected by healthcare providers
  - Insensitive care
  - Poor communication with healthcare team
  - Frustration with delays
  - Other
- Virtual care use
  - Decreased burden of in-person visits
  - Increased connectedness
  - Value of e-health apps
  - Other

**PANDEMIC'S IMPACT ON FC WELL-BEING**

- Physical Toll
  - Chronic and intense care demands
  - Inability to sleep
  - Physical demands from lifting and transfers
  - Other
- Mental Toll
  - Nonstop vigilance
  - Worsening stress and mental exhaustion
  - Guilt in asking for help
  - Role of being more than just a parent
  - Inability to engage in self-care
  - Feeling of isolation
  - Extreme fear of COVID-19
  - Other

**COMMON CHALLENGES DURING THE PANDEMIC**

- Financial strain
  - Significance of costs to meet care needs of CMC
  - Impact of caregiving on ability to work
  - Lack of funding and financial support
  - Significance of administrative work
  - Other
- Balancing multiple roles
  - Acting as a medical professional
  - Burden of coordinating care

    - Complex administrative work associated with care
    - Lack of family and homecare nursing support
    - Advocating for child’s needs
    - Impact on family dynamics
    - Other
- Inadequate homecare nursing
    - Challenges of frequent nurse turnover
    - Burden of scheduling nurses
    - Concerns with nurse competence
    - Negative experiences
    - Frustration with lack of improvement
    - Other
